# Demographics and Comorbidities of United States Service Members with Combat-Related Lower Extremity Limb Salvage

**DOI:** 10.3390/jcm12216879

**Published:** 2023-10-31

**Authors:** Stephen M. Goldman, Susan L. Eskridge, Sarah R. Franco, Christopher L. Dearth

**Affiliations:** 1 Research & Surveillance Division, DoD-VA Extremity Trauma and Amputation Center of Excellence, 8901 Wisconsin Ave., Bethesda, MD 20889, USA; 2 Department of Surgery, Uniformed Services University of the Health Sciences and Walter Reed National Military Medical Center, Bethesda, MD 20814, USA; 3 Leidos, Reston, VA 20190, USA

**Keywords:** trauma, abbreviated injury scale, military medicine, wound and injuries, amputation, musculoskeletal system

## Abstract

Introduction: This retrospective study describes the demographics and injury characteristics of a recently identified cohort of US Service members with combat-related lower extremity limb salvage (LS). Methods: US Service members with combat trauma were identified from the Expeditionary Medical Encounter Database and Military Health System Data Repository and stratified into primary amputation (PA), LS, and non-threatened limb trauma (NTLT) cohorts based on ICD-9 codes. Disparities in demographic factors and injury characteristics were investigated across cohorts and within the LS cohort based on limb retention outcome. Results: Cohort demographics varied by age but not by sex, branch, or rank. The mechanism of injury and injury characteristics were found to be different between the cohorts, with the LS cohort exhibiting more blast injuries and greater injury burden than their peers with NTLT. A sub-analysis of the LS population revealed more blast injuries and fewer gunshot wounds in those that underwent secondary amputation. Neither demographic factors nor total injury burden varied with limb retention outcome, despite slight disparities in AIS distribution within the LS cohort. Conclusions: In accordance with historic dogma, the LS population presents high injury severity. Demographics and injury characteristics are largely invariant with respect to limb retention outcomes, despite secondary amputation being moderately more prevalent in LS patients with blast-induced injuries. Further study of this population is necessary to better understand the factors that impact the outcomes of LS in the Military Health System.

## 1. Introduction

Extremity injuries constituted the majority of trauma experienced by United States Service members (SMs) during Operation Iraqi Freedom and Operation Enduring Freedom [1,2,3]. These types of injuries often involve multiple organ systems, adding complexity to their clinical care [4]. In many cases, the accumulated injuries pose a risk of limb loss, requiring a shared decision-making process between the patient and the clinical team to determine whether limb retention or immediate amputation is the preferred treatment strategy [5,6]. SMs who opt for limb retention often undergo multiple surgical procedures and intensive physical rehabilitation, collectively known as limb salvage (LS).

While the term “limb salvage” is commonly used, its precise definition has historically varied among providers. Consequently, conducting comprehensive epidemiological studies using large medical databases to assess the prevalence or incidence of LS in the context of lower extremity trauma has been challenging, and this population of SMs is understudied relative to other cohorts with readily identifiable medical codes (e.g., limb loss). As such, studies are often limited to studying a subset of limb salvage, as defined by either a narrow subset of injury types (e.g., Type III Gustilo Fractures [7], arterial injuries [8]) or a particular management plan (e.g., flap-based repair, vascular reconstruction) [9,10,11,12]. Subsequently, sample sizes are small, and interpretations are limited in scope.

This study aims to address this knowledge gap by utilizing a cohort of SMs with combat-related lower extremity (LE) LS, defined through a validated data-driven approach [13]. This study seeks to answer the following questions: (1) What are the demographic characteristics associated with the combat-related LS cohort? (2) What concomitant injuries are more frequently sustained by SMs who undergo LS? (3) Are there any correlated concomitant injuries that lead to secondary amputations?

## 2. Methods

### 2.1. Data Sources and Study Sample

This study was approved by the Naval Health Research Center (NHRC) Institutional Review Board and consisted of a retrospective database review of all combat-related injuries to lower extremities from 2004 to 2014 with an acute injury episode documented in the Expeditionary Medical Encounter Database (EMED; NHRC, San Diego, CA, USA) [14]. Inclusion criteria included the requirement of inpatient medical records within two years of the date of injury accessible within the Military Health System Data Repository (MDR). Exclusion criteria included a maximum lower extremity abbreviated injury scale (AIS) of one (i.e., minor trauma). Subsequently, an initial population of 4275 SMs with combat-related lower extremity trauma was identified. The initial population was then stratified into primary amputations (PA; i.e., amputation occurring ≤14 days after injury), non-threatened limb trauma (NTLT), and limb salvage (LS) cohorts using a combination of medical codes that has previously been reported to be significantly associated with limb salvage [13]. The identified LS cohort was further partitioned into those who went on to receive a secondary amputation (LS-SA, i.e., an amputation occurring ≥15 days after injury) and those who never underwent amputation (LS-NA). The PA and NTLT cohorts served as comparison groups.

### 2.2. Variables

Demographic variables, including age, sex, military branch, pay grade, the mechanism of injury, injury severity score (ISS), and maximum lower extremity abbreviated injury scale (AIS) were extracted from EMED records. The military branch was categorized as Army, Marine Corps, or other. Pay grade was categorized according to military rank: E1–E3, E4–E6, E7–E9, or Officer. Mechanisms of injury included blast, gunshot wound, or other. ISS was categorized based on severity mix as 1–4, 5–8, 9–15, 16–24, 25–49, or 50–75 [1].

Given the nature of combat-related trauma and the associated likelihood of injury to multiple body regions, especially in injury events due to explosions [2], the frequency of concomitant injuries was compared across cohorts. The concomitant injuries examined were selected a priori to include body regions and injury types that are characteristic of polytrauma and can influence recovery and rehabilitation following LS. Concomitant injuries were identified from initial injury coding from EMED using ICD-9-CM diagnosis codes (Table 1).

### 2.3. Statistical Analysis

Categorical variables are displayed as counts along with their respective percentages, while continuous variables are presented as the mean and standard deviation (SD). To compare continuous variables, we conducted *t*-tests, and for categorical variables, we utilized chi-square tests, followed by post hoc Fisher’s exact tests with Bonferroni correction, setting alpha at 0.05. All calculations were performed using IBM SPSS Statistics (Version 28.0.1.1, IBM Corp, Armonk, NY, USA).

## 3. Results

While there was a nominal difference (Table 2) in the age of the extremity trauma cohorts (*p* = 0.008), no differences were observed between groups with respect to sex (*p* = 0.569), branch of the military (*p* = 0.348), or pay grade (*p* = 0.317). The LS cohort is predominantly male (98.0%) with an average age of 25.6 ± 6.1 years. A total of 70.4% of the cohort is from the Army, while 26.6 belongs to the Marine Corps. The overwhelming majority of the cohort comes from the enlisted ranks. No difference in age, sex, branch, or pay grade was observed for the LS subgroups associated with limb retention outcome (Table 3). The mechanism of injury was found to be different (χ^2^ = 356.1, 6 DF, *p* < 0.001) between the cohorts, with the PA cohort exhibiting the highest prevalence of blast injuries (95.5%) and LS (79.3%) and NTLT (64.9%) exhibiting lower rates in a stepwise fashion. Subsequently, gunshot wounds (GSWs) followed the opposite pattern, with NTLT exhibiting the highest prevalence (31.3%), followed by LS (16.9%) and PA (2.0%). Further analysis of the LS subgroups revealed that the LS-SA cohort experienced a higher prevalence of blast injuries (89.2%) and a lower prevalence of GSWs (7.1%) relative to the LS-NA cohort (blast 77.8%, GSW 18.4%).

Injury severity score (ISS) also varied across the LE trauma cohorts both with respect to the population mean (*p* < 0.001) and distribution (*p* < 0.001). The mean ISS for the LS cohort was higher than those with NTLT (*p* < 0.001) but lower than their peers with PA (*p* < 0.001). When binned according to severity mix [15], it was found that SMs belonging to the LS cohort were more likely to have an ISS in the range of 4–8 or 9–15 than their peers with PA and less likely to fall into severity mixes of 16–24 or 25–49. Compared with the NTLT cohort, the LS cohort exhibited an ISS distribution skewed toward a higher severity mix. Notably, the NTLT cohort exhibited a higher prevalence of ISS scores in the 4–8 range (37.0% vs. 27.1%, *p* < 0.001), while the LS cohort exhibited a higher prevalence of ISS scores in the 9–15 range. No differences between groups were observed for higher-scoring bins. No difference in the mean ISS (*p* = 0.707) or severity mix was observed between the LS subgroups.

The distribution of the maximum LE AIS score was disparate between the LE trauma cohorts (χ^2^ = 1359, 6 DF, *p* < 0.001). While a maximum LE AIS score of two was most prevalent for the NTLT cohort, post hoc Fisher’s exact tests revealed that SMs from the LS cohort were more likely to have a maximum LE AIS of three (50.8% > 36.8%, *p* < 0.001) or four (4.4% > 1.7%, *p* < 0.001) relative to NTLT but less likely to have a maximum LE AIS score of four (29.3% > 4.4%, *p* < 0.001) or five (16.9% > 0.9%, *p* < 0.001) than the PA cohort. No difference was observed between LS and NTLT for maximum LE AIS scores of five. Among the LS cohorts, SMs from the LS-SA cohort were found to be less likely to have a maximum LE AIS score of two (34.2% < 45.3%, *p* = 0.014) and more likely to have a maximum LE AIS score of three (60.2% > 49.4%, *p* = 0.023) than the LS-NA cohort. No difference was observed between the two LS cohorts for maximum LE AIS scores of four or five. The prevalence of polytrauma also varied across cohorts, with PA exhibiting greater representation than LS (30.4% > 15.0%, *p* < 0.001). No difference in the prevalence of polytrauma was observed between LS and NTLT or between LS subgroups.

Analysis of co-occurring injuries revealed each of the injury patterns studied was disparately observed within the extremity trauma cohorts (Table 4). Relative to NTLT, the LS cohort exhibited a greater rate of fracture of the skull (Fisher’s Exact, *p* = 0.010) and lower rates of fracture of the spine and trunk (Fisher’s Exact, *p* < 0.001) and internal injury of the chest, abdomen, and pelvis (*p* = 0.010). Relative to PA, the LS cohort exhibited lower rates of internal injuries of the chest, abdomen, and pelvis (*p* < 0.001), open wounds on the head, neck, and trunk (*p* < 0.001), open wounds on the upper limbs (*p* < 0.001), injuries to blood vessels (*p* < 0.001), injuries to nerves and the spinal cord (*p* < 0.010), and burns (*p* = 0.030). No disparities in co-occurring injuries were observed between the LS subgroups (Table 5).

## 4. Discussion

The observations reported herein represent the demographic profile and concomitant injuries of a cohort of SMs who underwent combat-related LS, and the subgroups within it based on penultimate limb retention outcome. In accordance with the prior literature [16], the LS cohort was characterized by more severely injured extremities relative to the NTLT comparison group and a high degree of polytrauma, yet this cohort had less severe injuries and a lower degree of polytrauma relative to PA. This is likely explained by the relative prevalence of blast injuries among the extremity trauma cohorts, as it is well established that polytrauma is commonly seen as a result of explosive mechanisms [17] due to blast-related primary (results from blast wave through the body), secondary (results from flying debris), tertiary (results from being thrown by the blast), and quaternary (all other explosion-related injuries) injuries [18]. Among the concomitant injuries more prevalent within the LS population, vascular injuries affecting body regions exclusive of the lower extremities were found to exhibit the most disparate frequency. This disparity is also likely explained by the prevalence of the blast mechanism of injury in this group, as it has previously been reported that explosive munitions were commonly associated with penetrating vascular injury [19,20].

The NTLT cohort had a higher prevalence of internal injury of the chest, abdomen, and pelvis compared to the LS cohort (16.2% vs. 20.9%; *p* = 0.010). This observation has multiple plausible explanations. First, the disparity may be linked to the fact that gunshot wounds (GSWs) were the predominant mechanism of injury among the NTLT cohort. Evidence in the literature from civilian public mass shootings suggests a strong relationship between the number of GSWs and the number of fatal organ injuries. Moreover, the location of the GSW varied by body area, with the chest/upper back and extremities both exhibiting > 1 GSW per victim, representing a significantly higher prevalence than the head and neck regions [21]. Furthermore, it was also noted that the location of the fatal wound occurring in the extremity in these cases was rare. If we apply this knowledge from the civilian world to a military context, wherein the usage of body armor has been associated with a sizeable reduction in the number of fatal thoracic injuries, irrespective of the mechanism of injury, incurred during conflict situations [22,23], it is plausible that the higher prevalence of internal thoracic injuries observed within the NTLT cohort could be associated with behind armor blunt trauma (BABT), which is succinctly defined as a non-penetrating thoracic injury due to the rapid deformation of body armor impacted by a high-energy projectile [24]. Based on the dependence of BABT on energy transfer, it is unlikely that such injuries would occur as frequently via explosive mechanisms, as the kinetic energy of the blast fragments can be substantially lower than bullets owing to the size and spread of the projectiles as well as the distance of the victim from the explosion. Further regional analysis of non-fatal ballistic wounding patterns among combat-injured SMs is necessary to support this conjecture.

Further analysis of the LS cohort revealed that individuals who entered the LS treatment pathway but ultimately opted for or required treatment with amputation (i.e., secondary amputation, LS-SA) exhibited higher LE AIS scores of 3, whereas the cohort that did not experience limb loss (i.e., LS-NA) more often exhibited a maximum LE AIS score of 2. While it is possible, even likely, that the disparities in limb retention outcomes within the LS cohort are at least partially explained by the observed disparities in the wounding mechanism and resultant local injury burden between LS-SA and LS-NA, it is also plausible that there are differences in pathology and/or clinical care that have not yet been elucidated and require further study in order to move toward understanding what factors are correlated with or predictive of limb retention outcomes of a limb salvage patient.

## 5. Limitations

These results presented herein suggest that the combat-related LS population is indicative of a greater portion of highly complex cases than NTLT, as determined by AIS and ISS. However, importantly, there are inherent limitations to making inferences based on AIS and ISS. Specifically, ISS does not account for multiple injuries to the same body part [25]. Despite past modifications made to AIS to make it more applicable to combat injuries (i.e., AIS-2005-Military and AIS-2008-Military), significant drawbacks in using this scoring system to adequately address the complexity of injuries suffered by SMs remain [26]. This study addressed the limitations of AIS, in part, by characterizing the concomitant injuries sustained by SMs with LS.

Another limitation of this study is that it does not report specifics on the proximity of the associated vascular injuries to the lower extremity, nor are there details of the operative techniques used to repair the injured vessels (e.g., autologous grafts, bypass, and ligation). Furthermore, details on total limb ischemia time (if any) and interval to reperfusion, both of which are directly correlated with adverse events, are not reported, as they were outside of the scope of this study but warrant future investigation.

Finally, this study only investigated one surgical outcome of LS, namely limb retention. Future efforts will more comprehensively define this LS cohort in terms of rates of acquired secondary musculoskeletal health conditions and return to duty, as well as evaluate healthcare utilization patterns.

## 6. Conclusions

The aim of this study was to examine the demographics and associated injuries in a group of service members who underwent limb salvage procedures. As expected, the LS group had less severe injuries compared to the primary amputation group, but their injuries were more serious than those in the non-threatening limb trauma group. This difference is likely due to the higher incidence of blast-induced injuries in the cohorts. Within the LS subgroups, despite similar demographic characteristics, there were variations in the mechanisms of injury and injury severity. Those who ultimately required secondary amputation had higher rates of blast injuries and higher maximum lower extremity injury scores (AIS). This observation emphasizes the importance of considering injury mechanisms and severity when distinguishing LS from other SM groups with extremity injuries. Furthermore, our findings highlight the necessity for further research on the LS population to gain a better understanding of the factors that impact patient outcomes so as to (1) enhance tools for clinical decision making and (2) identify capability gaps in the development of next-generation diagnostics and therapies.

## Figures and Tables

**Table 1:** Definition of co-occurring injuries.

Injury Description	ICD-9 Code
Fracture of skull	800–804.3
Fracture of spine and trunk	805–809.1
Fracture of upper limb	810–819.1
Intracranial injury; excludes skull fractures	850–854.1
Internal injury of chest, abdomen, and pelvis	860–869.1
*Traumatic hemothorax/pneumothorax*	860
*Injury to heart/lung*	861
*Injury to other/unspec intrathoracic*	862
*Injury to GI tract*	863
*Injury to liver*	864
*Injury to spleen*	865
*Injury to kidney*	866
*Injury to pelvic organs*	867
*Injury to intra-abdominal*	868
*Other internal*	869
Open wounds on head, neck, and trunk	870–879.9
Open wounds on upper limb	880–887.7
Injury to blood vessels; excludes lower limb	900–903.9
*Head*	900
*Thorax*	901
*Abdomen/pelvis*	902
*Upper limb*	903
Injury to nerves and spinal cord; excludes LE	950–955.9
*Injury to optic nerve*	950
*Injury to other cranial nerves*	951
*Spinal injury without bone injury*	952
*Injury to nerve roots/spinal plexus*	953
*Injury to other nerves of trunk*	954
*Injury to upper limb nerves*	955
Burns	940–949.9

Note: Subordinate code descriptions are represented in italics.

**Table 2:** Demographics and injury characteristics by cohort designation.

Classifiers	PA N = 885	LS N = 2018	NTLT N = 1372	Adjusted *p*-Values		
				χ*^2^* Test or ANOVA	Fisher’s Exact	
					PA vs. LS	LS vs. NTLT
**Age (mean ± SD)**	24.9 ± 5.0	25.6 ± 6.1	25.4 ± 5.9	**0.008**	>0.999	>0.999
**Male (n (%))**	869 (98.2)	1977 (98.0)	1339 (97.6)	0.569		
**Branch (n (%))**				0.348		
*Army*	591 (66.8)	1421 (70.4)	942 (68.7)			
*Marine Corps*	260 (29.4)	536 (26.6)	386 (28.1)			
*Other*	34 (3.8)	61 (3.0)	44 (3.2)			
**Pay grade (n (%)) ^†^**				0.317		
*E1–E3*	261 (29.5)	562 (27.8)	386 (28.1)			
*E4–E6*	519 (58.6)	1076 (53.4)	762 (53.6)			
*E7–E9*	34 (3.8)	102 (5.0)	77 (5.6)			
*Officer*	67 (7.6)	126 (6.2)	77 (5.6)			
**Mechanism of injury (n (%))**				**<0.001**		
*Blast*	845 (95.5)	1601 (79.3)	891 (64.9)	**<0.001**	**<0.001**	**<0.001**
*Gunshot wound*	18 (2.0)	341 (16.9)	429 (31.3)	**<0.001**	**<0.001**	**<0.001**
*Other*	22 (2.5)	76 (3.8)	52 (3.8)	0.178		
**ISS (mean ± SD)**	20.1 ± 10.7	12.6 ± 8.8	11.8 ± 8.9	**<0.001**	**<0.001**	**<0.001**
**ISS categories (n (%))**				**<0.001**		
*1–3*	--	--	--	--	--	--
*4–8*	1055 (31.1)	547 (27.1)	508 (37.0)	**<0.001**	**<0.001**	**<0.001**
*9–15*	1531 (45.2)	979 (48.5)	552 (40.2)	**<0.001**	**<0.001**	**<0.001**
*16–24*	500 (14.7)	315 (15.6)	185 (13.5)	**<0.001**	**<0.001**	0.254
*25–49*	277 (8.2)	158 (7.8)	119 (8.7)	**<0.001**	**<0.001**	0.545
*50–75*	27 (0.8)	19 (0.9)	8 (0.6)	**0.038**	0.161	0.545
**Max lower extremity AIS (n (%))**				**<0.001**		
*1*	--	--	--	--	--	--
*2*	1708 (50.4)	885 (43.9)	823 (60.0)	**<0.001**	**<0.001**	**<0.001**
*3*	1531 (45.2)	1026 (50.8)	505 (36.8)	**<0.001**	0.113	**<0.001**
*4*	113 (3.3)	89 (4.4)	24 (1.7)	**<0.001**	**<0.001**	**<0.001**
*5*	38 (1.1)	18 (0.9)	20 (1.5)	**<0.001**	**<0.001**	0.136
Polytrauma (n (%)) *	269 (30.4)	302 (15.0)	212 (15.4)	**<0.001**	**<0.001**	0.697

Note: ^†^ Percent does not add up to zero due to missing data. * Polytrauma is defined as two AIS regions > 2. Statistically significant findings are indicated by bolded *p*-values. Subordinate classifiers are represented by italics.

**Table 3:** Demographics and injury characteristics of limb salvage population by outcome.

Classifiers	LS-SA n = 269	LS-NA n = 1749	Adjusted *p*-Values
**Age (mean (SD))**	24.8 ± 5.1	25.7 ± 6.2	0.187
**Male (n (%))**	266 (98.9)	1711 (97.8)	1.000
**Branch (n (%))**			
*Army*	176 (65.4)	1245 (71.2)	0.770
*Marine Corps*	81 (30.1)	455 (26.0)	0.981
*Other*	12 (4.5)	49 (2.8)	0.989
**Pay grade (n (%)) ^†^**			
*E1–E3*	90 (33.5)	472 (27.0)	0.548
*E4–E6*	141 (52.4)	935 (53.5)	1.000
*E7–E9*	12 (4.5)	90 (5.2)	1.000
*Officer*	22 (8.2)	104 (5.9)	0.988
**Mechanism of injury (n (%))**			
*Blast*	**240 (89.2)**	**1361 (77.8)**	**<0.001**
*Gunshot wound*	**19 (7.1)**	**322 (18.4)**	**<0.001**
*Other*	10 (3.7)	66 (3.8)	1.000
**ISS (mean (SD))**	13.8 ± 10.4	12.5 ± 8.5	0.707
**ISS categories (n (%))**			
*1–4*	0	0	--
*5–8*	59 (21.9)	488 (27.9)	0.661
*9–15*	142 (52.8)	837 (47.9)	0.962
*16–24*	44 (16.4)	271 (15.5)	1.000
*25–49*	17 (6.3)	141 (8.0)	1.000
*50–75*	7 (2.6)	12 (0.7)	0.169
**Max lower extremity AIS (n (%))**			
*1*	0	0	--
*2*	**92 (34.2)**	**793 (45.3)**	**0.014**
*3*	**162 (60.2)**	**864 (49.4)**	**0.023**
*4*	9 (3.3)	80 (4.6)	1.000
*5*	6 (2.2)	12 (0.7)	0.432

Note: ^†^ Indicates that percentages do not add up to 100 due to missing data. Statistically significant findings are indicated by bolding. Subordinate classifiers are represented by italics.

**Table 4:** Co-occurring injuries by lower extremity trauma cohort designation.

Injuries	PA N = 885		LS N = 2018		NTLT N = 1372		χ^2^ Test	Fisher’s Exact Test Adjusted *p*-Value	
	*f*	%	*f*	%	*f*	%	*p*-Value	PA vs. LS	LS vs. NTLT
Fracture of skull	98	11.1	225	11.1	107	7.8	0.003	>0.999	0.010
Fracture of spine and trunk	165	18.6	375	18.6	359	26.2	<0.001	>0.999	<0.001
Fracture of upper limb	321	36.3	406	20.1	232	16.9	<0.001	<0.001	0.183
Intracranial injury; excludes skull fractures	317	35.8	655	32.5	399	29.1	0.003	0.566	0.321
Internal injury of chest, abdomen, and pelvis	224	25.3	327	16.2	287	20.9	<0.001	<0.001	0.010
Open wounds on head, neck, and trunk	538	60.8	894	44.3	590	43.0	<0.001	<0.001	0.998
Open wounds on upper limb	493	55.7	1215	60.2	870	63.4	<0.001	<0.001	1.000
Injury to blood vessels; excludes LE	91	10.3	560	27.7	116	8.4	<0.001	<0.001	0.757
Injury to nerves and spinal cord; excludes LE	110	12.4	171	8.5	143	10.3	0.003	0.010	0.467
Burns	117	13.2	190	9.4	128	9.3	0.003	0.030	1.000

Note: Frequencies represent the number of service members with at least one of the indicated diagnoses.

**Table 5:** Co-occurring injuries by lower extremity trauma cohort designation.

Injuries	LS-SA		LS-NA N = 1749		Fisher’s Exact Test
	*f*	%	*f*	%	*p*-Value
Fracture of skull	32	11.9	193	11.0	>0.999
Fracture of spine and trunk	58	21.6	317	18.1	0.861
Fracture of upper limb	57	21.2	349	19.9	>0.999
Intracranial injury; excludes skull fractures	99	36.8	556	31.8	0.681
Internal injury of chest, abdomen, and pelvis	52	19.3	275	15.7	0.814
Open wounds on head, neck, and trunk	119	44.2	775	44.3	>0.999
Open wounds on upper limb	71	26.4	559	32.0	0.551
Injury to blood vessels; excludes LE	15	5.6	90	5.1	>0.999
Injury to nerves and spinal cord; excludes LE	22	8.2	149	8.5	>0.999
Burns	25	9.3	165	9.4	>0.999

Note: Frequencies represent the number of service members with at least one of the indicated diagnoses.

## Data Availability

All data supporting the findings of this study are available within the paper.
